# Supplementary material for: Causal factors affecting gross motor function in children diagnosed with cerebral palsy
Source: PLoS One. 2022 Jul 18;17(7):e0270121. doi: 10.1371/journal.pone.0270121 (PMC9292109; doi:10.1371/journal.pone.0270121)
Supplement: Attachment — Submitted filename: PLOS one review_2022.pdf [file pone.0270121.s002.pdf]

Thank you for submitting this manuscript aiming to overcome the bottleneck in enhance motor function in children with cerebral palsy. This is an important start for clinical practitioners to re-consider and implement efficacious intervention to support this population. I still have few suggestions and questions regarding the content and result interpretation.

1. The proposed causal model was based on clinical speculations regarding relationship between impairments and associated motor function and is somehow supported by the result of linear models. However, consider the nature of retrospective approach with cross-sectional data set, it still needs to be more conservative in claiming a cause-and-effect relationship, which may be more complicated than it is interpreted in the present manuscript. I still suggested to tone down the conclusions while adding more discussion regarding the potential bidirectional or mediation effect.

    - For example, whether spasticity might be the mediating variables between selective motor control and functional outcome although the effect size of spasticity is small. This may also lead to a different judgement, together with the current evidence, on whether common tone management approaches are still with their values.
    - *"The effect of age on gross motor function is accounted for both directly, reflecting motor learning associated with practice, and indirectly, reflecting neuromaturation and growth."* This is an example of not knowing the cause-and-effect relationship or uncertain direction of the influence of one factor on the other. For example, age can have both the positive and negative impact on gait and functional performance. The negative influence may come from increased body weight and severer secondary impairments such as contracture. The other example is bony torsion, which may be worsen by on developing bone with walking.

2. Introduction: I would also suggest introducing CP (the first few sentences of current 2nd paragraph) before getting into more details regarding the evidence of current intervention effects. Please also add more information regarding the effect in treating different impairments in children with CP in the introduction or discussion

3. Introduction: Functional training and environmental modifications have showed stronger evidence in improving motor function in children with CP. However, there is insufficient review on relevant evidence in the introduction. Thus, it is suggested to add this information and discuss how it may help to support your findings or vice versa

4. Introduction: The following sentence is suggested to be removed

    " This assumption is entirely plausible. In fact, we demonstrate below that strength is an important causal factor, though with a substantially smaller impact than bivariate estimates would suggest. "

5. Method: Are there references to support the proposed causal model? There is no reference in the whole section of “Causal model”

6. Method: Although addressed in the discussion, I still have concerned toward averaging bilateral data in children with unilateral involvement. The effect of independent variables may be offset with it. This may be worth to first test which side of the data shows stronger impact on the functional outcome or the difference between affected and non-affected side plays a better role. In addition, what is the proportion of children with hemiplegia?
